# Impact of climate change on non-communicable diseases due to increased ambient air pollution

**DOI:** 10.25646/11655.2

**Published:** 2023-09-06

**Authors:** Susanne Breitner-Busch, Hans-Guido Mücke, Alexandra Schneider, Elke Hertig

**Affiliations:** 1 LMU Munich Faculty of Medicine, Institute for Medical Information Processing, Biometry and Epidemiology (IBE) Munich, Germany; 2 Helmholtz Zentrum München – German Research Center for Environmental Health, Institute of Epidemiology Munich, Germany; 3 German Environment Agency Department for Environmental Hygiene Berlin, Germany; 4 University of Augsburg Faculty of Medicine Augsburg, Germany

**Keywords:** AIR POLLUTANTS, HEALTH, CARDIOVASCULAR AND RESPIRATORY DISEASES, AIR TEMPERATURE

## Abstract

**Background:**

The impacts of air pollutants on health range from short-term health impairments to hospital admissions and deaths. Climate change is leading to an increase in air pollution.

**Methods:**

This article addresses, based on selected literature, the relationship between climate change and air pollutants, the health effects of air pollutants and their modification by air temperature, with a focus on Germany.

**Results:**

Poor air quality increases the risk of many diseases. Climate change is causing, among other things, more periods of extreme heat with simultaneously increased concentrations of air pollutants. The interactions between air temperature and air pollutants, as well as their combined effects on human health, have not yet been sufficiently studied. Limit, target, and guideline values are of particular importance for health protection.

**Conclusions:**

Measures to reduce air pollutants and greenhouse gases must be more strictly implemented. An essential step towards improving air quality is setting stricter air quality limit values in Europe. Prevention and adaptation measures should be accelerated in Germany, as they contribute to climate-resilient and sustainable healthcare systems.

## 1. Introduction

In the course of their lives, people are exposed to different risk factors that can have a negative impact on their health. Some of these risk factors, such as smoking and physical inactivity, can be influenced by structural changes (e.g. bans on tobacco advertising, availability of cycle paths), but also by people’s behaviour (their individual lifestyles). Other risk factors, such as air pollution, on the other hand, can mainly be influenced by structural changes, e.g. by legally setting maximum permissible emissions. However, individual behavioural changes can contribute, e.g. by consciously choosing environmentally friendly mobility, energy, and heating systems.

According to the World Health Organization (WHO), about 99% of the world’s population live in areas where air quality standards do not meet recommended guideline values. According to the latest State of the Global Air report [1], which is based on the findings of the Global Burden of Disease (GBD) study [2], it is estimated that air pollution was responsible for one in nine deaths worldwide in 2019. Air pollution is thus one of the four most important risk factors for the global burden of disease, surpassed only by high blood pressure, smoking, and poor nutrition. Info box 1 introduces the main air pollutants in Germany and their sources.

When considering exposure in the light of the new WHO guideline value for PM_2.5_ of 5 μg/m³ (annual mean value) published in 2021, almost 100% of the German population are exposed to PM_2.5_ fine dust values above the guideline value (Info box 2). Based on the results of the GBD study, the burden of disease due to air pollution is the tenth most important risk factor for human health in Germany and is considered the most important environmental risk factor [2].

**Info box 1 Major air pollutants relevant to human health in ambient air in Germany**

**Particulate air pollutants:** These can be of natural origin or generated by human activities. An important source of particulate air pollutants is motor vehicle traffic. In addition to exhaust emissions, there is also the whirling up of dust, wear of brakes and tyres, and abrasion of road surfaces. Other important sources are chimneys of industrial plants and power stations, heating systems in private households as well as agriculture. Natural sources of particles are emissions from volcanoes and oceans, forest and bushfires, and certain biogenic aerosols such as viruses.

To enable a uniform classification, the particles present in the air are often divided into the following categories based on their aerodynamic diameter:

► Total Suspended Particles (TSP): mass of all particles contained in total suspended particulate matter, this value is no longer routinely measured and regulated
► Inhalable particulate matter: particles whose aerodynamic diameter is smaller than 10 micrometres (<10 μm, abbreviated PM_10_)
► Coarse PM: Particles whose aerodynamic diameter is <10 μm but larger than 2.5 micrometres (>2.5 μm, PM_10–2.5_)
► Respirable particulate matter: particles with an aerodynamic diameter <2.5 μm (PM_2.5_)
► Ultrafine particles (UFP): particles whose aerodynamic diameter is <0.1 μm or smaller than 100 nanometres (PM_0.1_)

Concentrations of larger particles (PM_10_, PM_10–2.5_, and PM_2.5_) are usually determined as particle mass. UFP, on the other hand, contribute very little to the particle mass, but conversely determine the number of particles in the ambient air. Therefore, the appropriate measurement parameter for UFP is their number per unit volume (e.g. number per cubic centimetre, cm^3^).

**Gaseous air pollutants:** O_3_, ground-level ozone, is not released by a pollutant source, but is formed as a result of complex conversion processes. These mechanisms mainly involve volatile organic compounds and nitrogen oxides; solar radiation provides the energy for the formation of ground-level ozone. Therefore, a particularly large amount of ozone is produced, especially in summer and in anthropogenically polluted air masses.

Nitrogen dioxide (NO_2_) is a trace gas in the atmosphere and is produced as a by-product of natural and anthropogenic combustion processes. The main sources in ambient air are combustion engines and furnaces (for coal, oil, gas, wood, waste). In addition to its role as a precursor substance for ozone, NO_2_ is also involved in the formation of particulate matter.

Carbon monoxide (CO) is a gas that is formed during the incomplete combustion of fossil fuels. It forms when too little oxygen is available during combustion processes. Road traffic and combustion plants are the main sources. Carbon monoxide is also present in tobacco smoke in significant quantities.

Polycyclic aromatic hydrocarbons (PAHs) are substances produced by incomplete combustion processes of organic materials (e.g. wood, coal, or oil) or in food (e.g. during grilling or frying). The main sources are industrial processes in mineral oil processing, coal chemistry, metal processing, or energy production.

Air quality is generally subject to weather and shows weather-dependent as well as seasonal and interannual fluctuations. The average concentrations of air pollutants show a clear urban-rural gradient for particulate matter (PM) and nitrogen oxides (nitrogen monoxide and nitrogen dioxide, NO_X_). The highest pollution levels occur near their point of origin, in urban areas and places with heavy traffic. Low-wind weather conditions and inversion weather types, characterised by a reversal of the usual temperature decrease with altitude, thus preventing vertical air exchange, can lead to a strong accumulation of air pollutants in the lower atmosphere. In contrast, precipitation events usually lead to a reduction of pollution through leaching processes. High ozone concentrations mainly occur in Central Europe in the spring and summer months, often in combination with high air temperature and strong UV radiation, as ozone is formed photochemically under solar irradiation (discussed in more detail in this status report by Baldermann et al. [3]). Therefore, UV-intensive high-pressure weather conditions are usually associated with high ozone levels and high temperatures [4]. Concentrations of ozone generally increase towards suburban and rural areas. The highest pollution levels are caused by chemical reactions of the precursors of ozone, nitrogen oxides and volatile hydrocarbons, usually outside urban areas at some distance from the sources. The precursors of ozone are transported out of the city by wind, where they have time to react to form ozone. In addition, in inner cities, much of the ozone is immediately broken down by reacting with nitrogen monoxide (NO) from car exhausts. This is why ozone pollution in inner cities, with lots of traffic, is significantly lower than on the outskirts and in neighbouring rural areas.

**Info box 2 What are WHO guideline values?**

A central task of the WHO is the scientific derivation and publication of guideline values for the protection of human health: guideline values are numerical values expressed as the concentration of a pollutant in a specific medium (e.g. air, water) with an averaging time (period for which the guideline value is valid). It is assumed that no or minimal adverse health effects occur below this concentration. This relies on the accuracy and safety of currently available physical, epidemiological, and medical measurement and analysis methods, which is why the occurrence of possible health effects below a guideline value cannot be excluded in principle at present. Guideline values are not legally binding. Rather, they are recommendations for the protection of human health.

Air pollutants and greenhouse gases are mostly different substances, but often have the same sources. The emission of greenhouse gases (especially carbon dioxide (CO_2_), methane, and nitrous oxide) is a major cause of global warming, which has a lasting negative impact on the environment and health [5, 6]. The increase in mean air temperature changes the atmospheric circulation, short-term weather patterns, and long-term climate. Changes in atmospheric transport and mixing processes influence physico-chemical processes and the state of air quality. Since the beginning of this century, it has been shown that extreme weather events, which impact air hygiene, have increased and intensified, especially during the summer months, in Europe and Germany. These include, in particular, periods of extreme heat with simultaneously increased concentrations of air pollutants such as ozone, which can trigger health effects [7, 8]. Due to the correlation of ozone formation processes with temperature, ozone concentrations are expected to increase as climate change continues, especially in projections with strong climate change and limited reduction of precursor substances. In addition, exposure to PM_10_, whose emissions may come from both anthropogenic and natural sources, may increase. The anthropogenic share is emitted by combustion processes in industry and traffic. Natural processes such as vegetation fires [9] and the dispersal of dusty and dry soil by wind during prolonged summer droughts, such as the drought summer of 2018 [10], can be a significant additional burden on total PM emissions.

This article provides an overview of the health effects of air pollutants and their interaction with air temperature, as well as an assessment of the relevant limit, target, and guideline values. Finally, recommendations for public health are collected. The analysis is based on the current literature within the framework of a narrative review.

## 2. Health effects of air pollutants

### 2.1 Overview

Although air pollutants primarily enter the body via the respiratory tract, which initially suggests a health risk for the respiratory tract and lungs, research over the past decades has shown that the greatest attributable risk of air pollutants lies with the cardiovascular system. The effects range from short-term health impairments to hospital admissions and deaths. These can occur acutely at high concentrations or as a consequence of long-term exposure [11].

**Figure 1:**
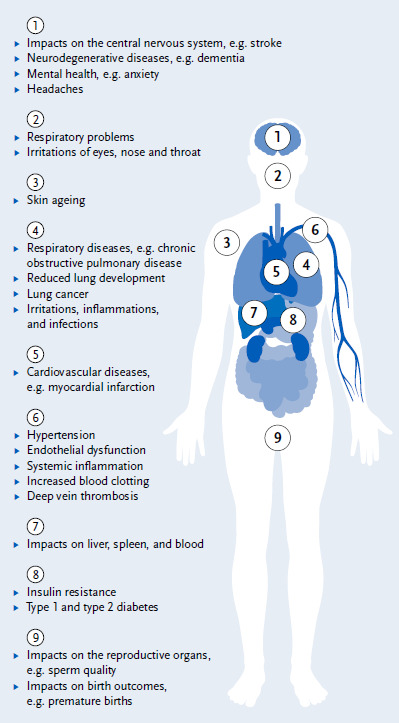
Health impacts of air pollutants Source: Own representation based on European Environment Agency [15] and Thurston et al. [12]

Poor air quality increases the risk of heart disease, lung disease and respiratory infections, type 2 diabetes, and other health problems (Figure 1). Exposure to air pollutants during pregnancy can lead to an increased risk of pre-term birth and low birth weight. Air pollutants have also been linked to asthma and lower respiratory tract infections in children. These impacts on health can lead to school and work absenteeism, chronic illness, and death. Overall, exposure to air pollutants shortens life expectancy [12–14]. Air pollution is by far the most important environmental risk factor for human health.

With regard to health impacts, a distinction is usually made between short-term effects of high air pollutant concentrations – effects that occur in the immediate temporal vicinity of exposure, i.e. within a few days – and those effects that result in the long term from increased (chronic) exposure to air pollutants (e.g. annual average exposures at the place of residence or work). Short-term increases in PM_2.5_ increase the relative risk of acute cardiovascular events by 1 to 3% within a few days. Longer-term exposures over several years increase this risk to a greater extent (about 10%), partly due to the development and/or exacerbation of cardiometabolic diseases such as hypertension and diabetes mellitus [16]. Reducing air pollution could significantly lower disease burden from strokes, heart disease, lung cancer, and both chronic and acute respiratory diseases, including asthma.

In particular, short-term elevated exposures may pose little risk to healthy individuals. However, subclinical health effects may well be considered a plausible precursor to serious or fatal events in susceptible individuals. In contrast, repeated exposures or high long-term exposure may contribute to the development of cardiovascular and respiratory diseases. The health impacts associated with air pollution may seem small from a medical point of view compared to other risk factors such as smoking or obesity, but on a population-wide basis the effects are immense.

Despite the good state of research, no lower threshold for health effects could be identified thus far. Even at low exposure levels, the health impacts increase with increasing air pollutant concentrations. This was investigated in a large study with several million participants in the United States amongst others. The results showed a linear exposure-response relationship between PM_2.5_ and mortality, even far below the current U.S. limit of 12μg/m³ [17]. This means that every reduction in exposure is associated with a health gain – both in the short and long term [18].

#### Potential mechanisms

Air pollutants are transported into the lungs via the respiratory tract. Particularly PM_2.5_ reaches the smallest airways and alveoli. UFP can also reach other organs via the bloodstream (Info box 3). In summary, the particle effects can be caused by various mechanisms that – alone or together – increase the risk for cardiovascular diseases and cardiovascular events in particular (Table 1) [16, 19–22].

Furthermore, exposure to air pollutants can lead to epigenetic changes, e.g. changes in DNA methylation (chemical changes in the basic building blocks of a cell’s genetic material), which are similar to those of epigenetic ageing [16, 21]. Studies have also shown that air pollutants can accelerate the so-called epigenetic ageing clocks and thus increase the difference between chronological age and methylation age, with a larger difference being associated with lower life expectancy and a higher risk of age-related diseases [16, 21].

**Info box 3 Penetration depth of air pollutants**

**Particulate air pollutants:** In line with their size, i.e. along their aerodynamic diameter, aerosol particles can penetrate the human organism to different depths. Particles with aerodynamic diameters >10 μm (coarse particles) are almost exclusively deposited in the upper respiratory tract (mouth, nose, throat) in healthy adults and consequently pose a low risk. In contrast, the PM_10_ fraction can penetrate the upper part of the lungs and is therefore also referred to as inhalable fine dust. The even finer particles with an aerodynamic diameter <2.5 μm (PM_2.5_) reach the bronchi and bronchioles. Therefore, PM_2.5_ is also referred to as respirable particulate matter. UFP can penetrate the site of gas exchange (alveoli) and are able to pass into the bloodstream, which distributes oxygen throughout the human organism. These particles can thus be transported to different organs of the body [20].

**Gaseous air pollutants:** All gaseous air pollutants, such as sulphur dioxide, nitrogen oxides, and carbon monoxide penetrate the entire respiratory tract and stress the mucous membranes there. Ozone, in particular, can reach the deepest parts of the lungs if inhaled deeply and frequently.

The pathways and mechanisms shown in Table 1 are partly interdependent and can cross-react (e.g. ‘feed-forward’ or mutual reinforcement). Overall, it is assumed that direct effects of particles can trigger cardiovascular events within a few hours. In addition, there is increasing evidence that particles contribute to the development and progression of atherosclerotic lesions, a possible mechanism for the observed long-term effects [20].

#### Susceptible population groups

Potential factors for particular susceptibility to air pollutants are age (infants, children, and older persons), smoking and other lifestyle factors. In particular, social status is a factor that plays an important role in the study of health impacts through air pollution. It correlates with other social and personal factors as well as with the environmental conditions at the place of residence. Specific life stages such as pregnancy also contribute to increased susceptibility, affecting both expectant mothers and the unborn. Chronic pre-existing conditions are another important factor: sensitivity is increased especially in children and older persons with chronic respiratory diseases (e.g. bronchial asthma, chronic obstructive pulmonary disease) and with cardiovascular diseases, and is reflected in sudden exacerbation of the underlying diseases on individual days, especially during episodes with high ambient air pollution (e.g. smog situations) [20, 23].

### 2.2 Health impacts of air pollutants in interaction with high air temperature

As mentioned above, high air temperature combined with intensive solar radiation favours the formation of ground-level ozone through the reaction of nitrogen oxides and volatile organic compounds. In addition, particulate matter pollution can increase due to the formation of so-called secondary aerosols [24]. On hot days, there is also little air circulation, so air pollutants generated in cities, in particular, cannot be removed and remain in the air in higher concentrations. During hot spells with prolonged drought, forest fires may occur frequently, contributing significantly to high concentrations of pollutants, especially particulate matter [25].

Ozone and particulate matter (PM_10_ und PM_2.5_) are, therefore, of particular relevance to health during dry, hot, high-pressure weather conditions in summer. Health-related studies have indicated the influence of air pollutants when heat co-occurs, mainly affecting people in urban areas [26, 27]. The exposure-dependent nature of health effects (parallel individual effects vs. combination effects on morbidity and mortality) cannot yet be conclusively assessed due to effect modification and the interaction of individual factors and thus requires further research [5, 26].

So far, high air temperatures or heat events and air pollutants have mostly been considered separately. Most studies to date have examined the health effects of different air pollutants by considering air temperature as a potential confounding factor and vice versa. However, the interactions between high air temperatures, heat events, and air pollutants, as well as their combined effects on humans, have not yet been sufficiently explored, especially considering global climate change [28, 29].

#### Effects of short-term exposure to air pollutants on mortality – effect modification by heat

Most studies published so far have examined the change in the effects of air pollutants due to temperature. The majority of these studies have shown that high temperatures increase the effects of ozone or particulate matter on (cause-specific) mortality. However, some studies also indicate stronger effects of ozone and particulate matter at lower temperatures, or show no change in the effect of air pollutants corresponding to temperature [30].

#### Effects of short-term exposure to heat on mortality – effect modification by air pollutants

In contrast, there are only a limited number of studies investigating whether air pollution modifies the effects of temperature [29, 31]. For example, in the aftermath of the hot summer of 2003, the independent and synergistic effects (i.e. a joint effect greater than the sum of the individual effects) of heatwaves and air pollutants on daily mortality in nine European cities were investigated [8] (see also Winklmayr et al. [32] in this progress report). It was shown that the mortality risk from heat was increased by simultaneously elevated concentrations of ozone and PM_10_. Older persons were particularly at risk. A recent systematic review with meta-analysis found similar results: a significant change in the heat effect on all-cause or natural mortality due to ozone and PM_10_ was observed, with stronger heat effects on days with high exposure to air pollutants [31]. These effects were confirmed by a comparative study carried out in eight German cities on temperature-dependent threshold value determination. According to this study, the ozone effect on total mortality appears to be greater at low air temperatures, whereas the temperature effect dominates equally as much as ozone during heat [33].

Another European study also showed that high air temperatures modify the effects of air pollutants on total mortality from natural causes and on mortality from cardiovascular diseases, and that high concentrations of particulate matter, UFP, and ozone amplify the effect of air temperature [6].

#### Combined effects of short-term exposure to air pollutants and heat on mortality

A U.S. study recently showed that the risk of death in California increased by about 6% on days with extremely high temperatures and by about 5% on days with high PM_2.5_ concentrations from 2014 to 2019 [34]. On days with both extreme heat and high air pollution, the risk increased by 21%, higher than the sum of the individual effects of extreme temperature and PM_2.5_ alone. Katsouyanni et al. [35] also found that high air temperature increased the adverse health impact of PM_10_; in a region with a warm climate, a 10 μg/m³ increase in particulate matter caused a 0.8% increase in all-cause mortality, whereas in a cooler climate, the increase was only 0.3%.

#### Short-term exposure to air pollutants and heat – effects on morbidity

The vast majority of studies conducted to date have examined the effects of the interaction of air temperature and air pollutants on mortality. In contrast, only a few studies have examined interactive effects or changes in effects on hospital admissions or other morbidity endpoints. For example, studies from Australia, China, and the United States have shown that high particulate matter concentrations increased the effects of heat on cardiorespiratory hospital admissions and that high temperatures influenced the effects of particulate matter; particle effects were generally stronger in heat [36]. A recent review with meta-analysis on respiratory hospital admissions also showed stronger PM_10_ particulate matter and ozone effects at concurrent high temperatures [37]. However, other studies showed stronger effects of air pollutants at concurrent lower temperatures or no interaction or effect modification [36].

Other studies also found evidence of interactive effects of temperature and particulate matter, soot, or ozone on lung function, heart rate and heart rate variability, blood pressure and markers of endothelial function [29].

#### Interaction of chronic exposure to air pollutants and air temperature

While an increasing number of papers in recent years have investigated the health effects of the association between short-term exposure to elevated air temperature and air pollutants, there have been very few studies on the interaction of chronic exposure to air pollutants and air temperature. However, given the changing climate, it is important also to understand the longer-term effects, such as annual average temperatures, and their interaction with chronic exposure to air pollutants.

A study of the association between chronic particulate matter pollution and mortality in 207 American cities showed that the PM_2.5_ effects were particularly pronounced in cities where the annual average temperature was higher [38]. Similar effects were described in other U.S. studies [29].

## 3. Limit, target, and guideline values with regard to the current air quality situation in Germany

Guideline values represent recommendations by experts from the relevant fields and are based on the current state of knowledge on the health effects of air pollutants (see WHO air quality guidelines), while limit values are laid down in ordinances and regulations. In addition, there are target values that should be met within a certain period of time. The legal basis for the protection of human health through air pollution control and air quality assessment is summarised in Info box 4. For PM_10_ the limit value is 50 μg/m^3^ as a daily mean (35 permissible exceedances per year) and 40 μg/m^3^ as an annual mean. For PM_2.5_ the annual mean limit value is 25 μg/m^3^. For ultrafine particles, which are also relevant to health, there are currently neither guideline nor target or limit values. For NO_2_ the limit values are 200 μg/m^3^ as an hourly average (18 permissible ex-ceedances per year) and 40 μg/m^3^ as an annual mean. For ozone, the daily maximum eight-hour mean may exceed 120 μg/m^3^ on a maximum of 25 days per calendar year, averaged over three years. In the long term, the maximum eight-hour mean should not exceed 120 μg/m^3^ at all.

Based on numerous new scientific studies, the WHO in 2021 revised its previous air quality guideline from 2005 and published it with new, global recommendations and guideline values [39]. Guideline values of 45 μg/m^3^ (daily mean) and 15 μg/m^3^ (annual mean) were derived for PM_10_, and 15 μg/m^3^ (daily mean) and 5 μg/m^3^ (annual mean) for PM_2.5_. For NO_2_, the new WHO guideline recommends a guideline value of 25 μg/m^3^ for the hourly average and 10 μg/m^3^ for the annual mean. The guideline values for ozone are 100 μg/m^3^ for the daily maximum eight-hour mean and a maximum of 60 μg/m^3^ as the highest six-month running-average.

**Figure 2:**
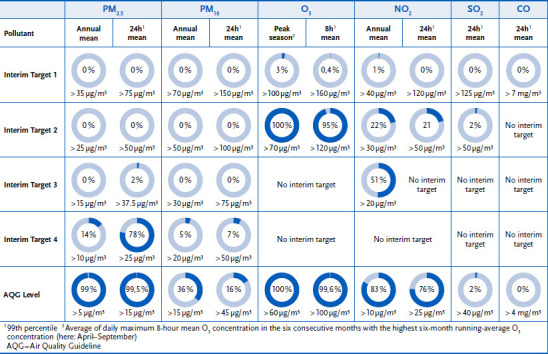
Share of air quality monitoring stations in Germany exceeding in 2020 (dark blue parts of the ring) the new WHO air quality guideline values and interim targets (2021) Source: Own representation based on Wichmann-Fiebig et al [40]

Due to the different levels of air pollution control in various countries around the world, interim targets were also recommended, through which a gradual development process should be initiated until the WHO guideline values are reached. It follows from the recommendations of the new WHO air quality guidelines that the concentrations of air pollutants currently measured in Germany and the limit values still in force are set too high to ensure effective health protection for the population. Figure 2 shows the percentage of exceedances of the WHO guideline values and interim targets for particulate matter (PM_10_ and PM_2.5_), ozone, NO_2_, SO_2_, and CO at the air quality monitoring stations in Germany in 2020. Various measures have been taken in Germany in the past to reduce air pollutant concentrations. The focus is on the desired reduction and avoidance of harmful effects on human health and the environment caused by air pollutants. Compliance with the prescribed air quality values and emission ceilings is intended to reduce pollution, although compliance with the legal limits does not mean complete health protection. All EU member states are obliged to draw up air pollution control and action plans in accordance with EU law in the event of exceedances of the EU air quality limit values from 2008. The results of the current air quality evaluation 2021 show that, for Germany, air pollution by particulate matter, NO_2_, and ozone must be further reduced on a large scale in order to protect public health [3].

**Info box 4 Legal basis for air pollution control and air quality assessment**

The legal basis for air pollution control and air quality monitoring has been created by international agreements, directives at European level and their transposition into German law. The member states of the European Union (EU) have developed uniform regulations for the assessment and control of air quality. The basis for this is Directive 2008/50/EC on ambient air quality and cleaner air for Europe of May 2008. The 39^th^ Ordinance (39^th^ BImSchV – Ordinance on Air Quality Standards and Emission Ceilings of August 2010) to the Federal Immission Control Act (BImSchG - Act on the Prevention of Harmful Effects on the Environment caused by Air Pollution, Noise, Vibration and Similar Phenomena) transposed the EU Directive into German law. Binding limit and target values have been set for various air pollutants. To ensure comparability of the measurements carried out in the individual member states, the directive contains binding regulations on the location and minimum number of sampling points, uniform criteria on data quality objectives and calculation rules, and specifications for the report of the air quality assessment to the EU Commission. Reference methods for assessing the various pollutant concentrations are also laid down here. Each member state reports to the EU Commission on September 30 of each year on the air quality of the previous year.

Source: Wichmann-Fiebig et al. [40].

## 4. Significance for public health and recommendations

Climate projections show that climate change is very likely to lead to an increase in extreme weather events and changes in air pollutants with different impacts on human health in the coming years and decades (see Hertig et al. [41] in this status report). Therefore, international agreements, national laws, and ambitious regulations must be increasingly implemented and adhered to, such as the measures to reduce greenhouse gas emissions of the German Climate Change Act as well as the new regulations on air pollution control and the reduction of air pollutant emissions, which are being revised. In addition, in the upcoming Climate Adaptation Act for Germany, which is currently being drafted, nationwide preventive and adaptation measures are prepared in order to enable and ensure effective health protection as well as early and precautionary adaptation of the population.

At the level of individual behavioural prevention, in order to avoid health burdens caused by air pollutants, the population should refrain from prolonged physical exertion at times of day that are associated with increased concentrations of air pollutants. This applies particularly to at-risk individuals with health problems, for example during the midday and afternoon hours when ozone concentrations are elevated. From the point of view of preventive health protection, an integrated environmental, economic, transport, climate and air pollution control policy should, in the sense of the ‘Health in All Policies’ approach, ensure sustainable compliance with the upper limits of the concentration values for air pollutants – at least the air quality values stipulated by law, but ideally the scientifically derived guideline values of the new WHO air quality guidelines 2021. Appropriate measures should prevent the uncontrolled increase in industrial and individual energy consumption and the associated rise in CO_2_ emissions. The release of ozone precursors, which are increasingly emitted in summer due to the use of air conditioning systems, should also be prevented.

### 4.1 Synergies of environmental, climate, and health protection

The use of fossil fuels such as coal, oil, and natural gas not only has a harmful effect on the climate, but also leads to air pollution that is harmful to health. We must systematically and quickly reduce greenhouse gas emissions and implement extensive energy-saving measures, while also switching to renewable energies to cover energy demand, thus reducing health risks from air pollution [42]. Large energy consumers in the health sector, such as inpatient healthcare facilities, run 24/7 and, together with outpatient service provision, cause an annual raw material consumption of about 107 million tonnes (half of which is biomass and fossil fuels), with about one-third coming from domestic raw material extraction and two-thirds from imports [43]. Implementing structural prevention measures for climate protection (mitigation), such as the energy renovation of hospitals and their replacement by new low-energy buildings adapted to climate change, would go a long way towards active mitigation and thus also improve health protection for patients and staff in these facilities. Patient care is the top priority and therefore, in view of increasing extreme heat events, adaptation measures for structural and air-conditioning renovation, shading, passive building cooling and, if medically necessary, individual room air-conditioning powered by renewable energies must be implemented. So far, there are no central and coordinated mitigation measures for a nationwide energy efficiency upgrade of existing hospital buildings.

An integrated environmental, climate and health policy alongside individual health behaviour oriented towards climate protection can have synergistic effects and generate win-win situations or health co-benefits of climate protection and climate adaptation measures. The following are examples of combined structural and behavioural prevention measures that aim to generate such co-benefits:

(1) Mobility: On separate and secured transport routes, cycling and other forms of active transport through physical activity not only avoid air pollutant emissions from motor vehicle traffic, but also reduce the risk of cardiovascular diseases as a co-benefit and promote fitness and health.

(2) Urban development: Urban development measures such as the deconstruction of sealed surfaces and the nature-orientated expansion of urban green spaces improve air quality and also reduce the risk of heat-related health problems through cooler air and shade. They serve the ecological needs of a city as well as the individual’s physical and mental health [44].

### 4.2 Climate-resilient and sustainable health systems

According to current estimates, the German health sector is responsible for 5 to 6.7% of Germany’s overall greenhouse gas emissions [45, 46]. About one-third of these are caused by emissions from heating and energy consumption of healthcare facilities and about two-thirds by upstream and downstream processes, such as the production of medical devices and pharmaceuticals, delivery processes, and waste disposal. Thus, the health sector is called upon to permanently minimise its greenhouse gas emissions as a climate protection and air pollution control measure while maintaining the same quality of basic care and high-quality standard of the services provided. However, Germany has so far lacked a national climate strategy for the health sector. A representative survey on the status of the transformation towards a climate-neutral and climate-resilient healthcare system asked managers and medical specialists about their personal attitudes and the implementation of environmental, climate protection, and sustainability measures, as well as barriers to implementation [47]. The survey showed that many decision-makers in the health sector are aware of the relevance and urgency of the issue. The vast majority of the physicians and managers interviewed agree that measures must be taken to address climate change in healthcare facilities. However, there is a lack of specialised knowledge of climate protection and sustainability, as well as clear accountability at the management level. An important factor for the implementation of the transformation of the healthcare system concerns the avoidance of unnecessary therapies, which will free up personnel and ecological resources. Furthermore, the study identified a lack of knowledge concerning concrete climate adaptation strategies, for example with regard to protection against summer heat in hospitals as part of internal alert planning and increasing resilience against extreme weather events. As part of the critical infrastructure, the health sector is only just beginning to face the enormous challenges in identifying its adaptation needs and strengthening its resilience in the long term [45].

The increased occurrence of climate change-associated physical and mental illnesses, for example triggered by extreme weather events, poses new challenges for those working in the health professions. The importance of climate change as a factor influencing health is not adequately reflected in the education, training, and continuing education of the health professions so far. It is recommended that further training regulations be adapted and supplemented to include appropriate content, enabling those working or planning to work in the healthcare sector to react appropriately to climate-related changes [45].

## 5. Discussion and conclusion

The air hygiene situation in Germany has improved significantly in recent decades. However, many cities and regions have not yet succeeded in complying with the European limits for air pollutant loads. Current studies predict that, despite air pollution control efforts, health risks from air pollution will continue to increase in the future, especially in conurbations and inner cities [25]. The reason for this is that energy consumption based on fossil combustion processes is still high or even increasing. Furthermore, a warmer climate can indirectly change the emissions of air pollutants and their precursors. In addition to increasing ozone formation potential, more frequent droughts can also increase particulate matter pollution, e.g. when they promote forest fires or through the deflation of parched soils. Besides these air pollutants, changes in the duration and intensity of pollen exposure could cause an additional health burden for people with allergy-related respiratory or lung diseases [48, 49]. Interactions with air temperature, specifically during heatwaves, may also influence the health effects of air pollutants.

Tightened limits for air pollution in the EU are essential to improving air quality in Europe. Lowering the values, especially for particulate matter and NO_2_, would be an important contribution to reducing the population’s disease burden due to air pollutants. It is also essential to expand the monitoring of air pollutants, both regulated and non-regulated, such as UFP, soot particles, and ammonia. A commitment to continuous improvement of air quality up to or below the guideline values of the new WHO air quality guidelines (2021) could maximise health benefits for the European population. Air pollution remains one of the leading causes of illness and death, and the burden of disease caused by air pollution in Europe is high. Among other things, this also poses a huge financial burden and puts strain on healthcare systems across the EU. Every single person would benefit from cleaner air, with babies, children, pregnant women, older persons, and people with cardiovascular and respiratory diseases among the most vulnerable groups. Therefore, we need an ambitious air quality regime that promotes and supports action at all levels – EU, national, local – and in all sectors such as transport, energy, industry, agriculture, and residential heating. The proposals initiated by the publication of the new WHO guideline, currently under discussion, to reduce EU limits and targets and to further improve current air quality contain important steps towards achieving cleaner air. However, greater ambition is needed to maximise health benefits for all. In addition, better air quality will help to mitigate the impacts of climate change and the associated health effects. It is, therefore, even more important that the EU values currently under discussion are achieved across the board before 2030.

The interaction of high air temperatures with heat events and air pollutants as well as their combined health effects have not yet been sufficiently researched, especially taking climate change into account. Therefore, in addition to the development of appropriate adaptation measures, further basic research on the combined effects of air pollutants and temperature on health is needed to gain a better understanding of the interrelationships. The effects of other multi-exposures, such as air pollutants and thermal exposure in combination with pollen and UV exposure, have also been insufficiently researched so far. Since atmospheric environmental exposures do not affect humans in isolation, but humans are instead exposed to a mix of environmental factors, a more comprehensive view is essential.

In the area of prevention and adaptation measures, air pollutants, temperature exposure, pollen, and UV should also be considered together in order to design and implement effective action measures. This applies to behavioural prevention measures as well as to the area of structural prevention. For example, heat-health action plans should not only address the thermal burden, but also include protective measures against anthropogenic and biogenic air pollutants. When designing urban green spaces, air quality and other factors for maintaining the mental and physical health of the urban population, such as accessibility and quality of stay should also be considered, in addition to the goal of reducing thermal load. In the health sector, a national climate change strategy should be developed that takes into account both the occupational health interests of all staff and the concerns of patients, but also considers changes in air pollutant exposures in the population. This can only succeed within the framework of inter- and transdisciplinary cooperation. Programmes that reduce air pollution lead to large health benefits that are compounded over time. The projected cost savings of health benefits from improved air quality far outweigh the implementation costs of air quality measures [50].

**Corrigendum**

On page 108 the process for the formation of ground-level ozone was described incorrectly. The precursors are nitrogen oxides and volatile organic compounds. The corrected sentence reads:

‘As mentioned above, high air temperature combined with intensive solar radiation favours the formation of ground-level ozone through the reaction of nitrogen oxides and volatile organic compounds.’ The article has been corrected accordingly.

## Key statements

- As climate change progresses, the health risks from air pollution could increase further.
- Ambient air pollution is one of the greatest environmental health risks.
- Interactive effects of air pollution and temperature need to be considered.
- Decision-makers should use synergies between environmental, climate, and health protection.
- The health sector is called upon to permanently reduce its greenhouse gas emissions as a climate protection and air pollution control measure.
- Reducing air quality limit values is essential to improving air quality in Europe.

## Figures and Tables

**Table 1:** Possible mechanisms that explain the observed relationship between air pollutants and disease Source: Own representation based on Schulz et al. [23]

Primary paths ➔➔➔	Mechanisms ➔➔➔	Possible consequences
Inhaled particles cause persistent oxidative stress and defence processes in the form of weak chronic inflammatory reactions	Release of messenger substances in the lung tissue, which leads to a systemic inflammatory reaction (involving the innate and acquired immune defence)	Disruption of endothelial function; formation of thrombi; progression of atherosclerotic lesions; impaired lung function; exacerbation of asthma and COPD; DNA damage; promotion of carcinogenesis and metastasis
Respirable particles or secondary mediators stimulate reflex receptors on the surface of the alveoli	Imbalance of the autonomic nervous system and thus influence on the autonomic control of the heart by favouring sympathetic tone via afferent nerve pathways; oxidation; alteration of central signalling pathways of cell differentiation and growth; mitochondrial dysfunction	Influence on cardiac output; cardiac arrhythmias; bronchoconstriction; damage to the mucous membrane of the respiratory tract; restriction of the self-cleansing mechanism of the bronchial tubes
Direct translocation: UFP or particle components penetrate the alveoli and enter the bloodstream	Direct influence on organs or blood components	Influencing the viscosity of the blood; local inflammatory reactions: increased inflammation levels and increased tendency to clot; central nervous system: effects on metabolism and hypothalamic-pituitary-adrenal axis activation

COPD=chronic obstructive pulmonary disease, DNA=deoxyribonucleic acid, UFP=ultrafine particles
