# Corrigendum: Impact of climate change on non-communicable diseases due to increased ambient air pollution

**DOI:** 10.25646/11831

**Published:** 2023-09-06

**Authors:** S Breitner-Busch, HG Mücke, A Schneider, E Hertig

Breitner-Busch S, Mücke HG, Schneider A, Hertig E (2023)

Journal of Health Monitoring 8(S4): 103–121.

DOI 10.25646/11655

On page 108 the process for the formation of ground-level ozone was described incorrectly. The precursors are nitrogen oxides and volatile organic compounds. The corrected sentence reads:

‘As mentioned above, high air temperature combined with intensive solar radiation favours the formation of ground-level ozone through the reaction of nitrogen oxides and volatile organic compounds.’ The article has been corrected accordingly.

The corrected version of the article is available at DOI 10.25646/11655.2

